# *FosB* mRNA Expression in Peripheral Blood Lymphocytes in Drug Addicted Patients

**DOI:** 10.3389/fphar.2018.01205

**Published:** 2018-10-24

**Authors:** Quézia Silva Anders, Jaisa Klauss, Livia Carla de Melo Rodrigues, Ester Miyuki Nakamura-Palacios

**Affiliations:** ^1^Laboratory of Cognitive Sciences and Neuropsychopharmacology, Program of Post-Graduation in Physiological Sciences, Health Sciences Center, Federal University of Espírito Santo, Vitória, Brazil; ^2^Laboratory of Neurotoxicology and Psychopharmacology, Program of Post-Graduation in Physiological Sciences, Federal University of Espírito Santo, Vitória, Brazil

**Keywords:** *FosB*, mRNA expression, lymphocyte, crack-cocaine use disorder, alcohol use disorder

## Abstract

*FosB* gene heterodimerizes with Jun family proteins to form activator protein 1 (AP-1) complexes that bind to AP-1 sites in responsive genes to regulate transcription in all cells. The genic expression of *FosB* seems to be modified after long time exposure to drugs of abuse and these changes may be involved in craving and addicted behavior. This study investigated the *FosB* mRNA expression in peripheral blood lymphocytes of drug addicted patients using real-time PCR approach. Thus, patients with crack-cocaine use disorder (CUD, *n* = 10), alcohol use disorder (AUD, *n* = 12), and healthy non-addicted subjects (CONT, *n* = 12) were assessed. *FosB* mRNA expression was reduced by 1.15-fold in CUD and 2.17-fold in AUD when compared to CONT. Hedge’s effect size g_s_ over log *FosB*/Act was of 0.66 for CUD and of 0.30 for AUD when compared to controls. This study showed that *FosB* mRNA expression was detected in lymphocytes from peripheral blood for the first time, and it was less expressed in drug addicted patients. This molecular technique may constitute a potential peripheral marker for substance use disorder.

## Introduction

The use of addictive drugs has been associated with alteration of gene expression in the brain that result in long-term changes on synapses and neural circuits and consequent neuroadaptive and behavioral changes such as tolerance and craving, which may underlie the development and maintenance of drug addiction (Nestler, 2008; Gajewski et al., 2016).

*FosB* gene heterodimerizes with Jun family proteins to form activator protein 1 (AP-1) complexes that bind to AP-1 sites in responsive genes to regulate transcription in all cells. There is evidence that the *FosB* genic expression can be modified after long-term drug exposure, increasing craving and aggravating the addictive behavior (Nestler, 2008; Gajewski et al., 2016).

According to Nestler (2012), drugs of abuse actives excitatory synapses increasing Ca^2+^ channels permeability into the neuron, triggering intracell targets to induce up or down regulation of genic expression. These alteration of genic expression in target genes like *CREB*, *BDNF*, and *FosB* would be involved in the development of a “state of addiction” (Nestler, 2013).

A truncated product of the *FosB* gene, the delta-*FosB*, gradually accumulates trough a course of repeated exposure to virtually all drugs of abuse and because of its unusual stability, its levels persist for weeks after drug cessation, mediating the sensitized responses to drug exposure (Nestler, 2008, 2013). Most studies focusing on molecular biology in drug addiction was conducted in rats (Robison and Nestler, 2012). There are no similar studies that has been done in the living brain of human drug addicts yet because of technical limitations. However, recently it has been suggested that the genic expression in peripheral blood lymphocytes may match to their expression state in the brain (Roozafzoon et al., 2010).

In this study, we analyzed *FosB* mRNA expression in peripheral blood lymphocytes (PBLs) of patients with crack-cocaine and alcohol use disorders in comparison to non-addicted subjects. To our knowledge this is the first study investigating the *FosB* gene expression in lymphocytes from peripheral blood as a potential marker for drug addiction condition.

## Materials and Methods

### Subjects

All subjects were informed about the purposes of the experiment by the principal investigator and signed a written consent before entering the study.

Ten patients, who met DSM V criteria for cocaine (crack) use disorder (CUD) and twelve alcohol use disorder patients (AUD) of both genders were successively recruited between October of 2017 and June of 2018 from a public hospital specialized in drug dependence treatment from Espírito Santo State, Brazil. They all received standard treatment provided by this hospital, consisting of psychosocial approaches – conducted by a professional team of psychologists, nurses, social workers, and physicians. They were not using pharmacological medications by the time the blood samples were collected for this study.

The control group was constituted by twelve healthy non-addicted and aged-matched subjects of both genders, recruited among workers from the University Hospital from Federal University of Espírito Santo and Hospital of Military Police of Espírito Santo. They were screened for drug use and included when were completely abstainer for alcohol and/or cocaine (Table 1).

**Table 1:** Socio-demographic characteristics and *FosB* expression values of the healthy non-user controls (CONT, *n* = 12) subjects, crack-cocaine use disorder (CUD, *n* = 10), and alcohol use disorder (AUD, *n* = 12) patients.

		Groups				
		CONT (*n* = 12)	CUD (*n* = 10)	AUD (*n* = 12)		*P*-value
*Socio-demographic characteristics*						
Age *[mean (SD)]*		47.9 (11.2)	42.5 (12.4)	42.3 (9.0)	*F*(2,33) = 1.0	*0.38*
Gender *n* (%)	Male Female	8 (66.7%) 4 (33.3%)	8 (80.0%) 2 (20.0%)	5 (41.7%) 7 (58.3%)	*X*_2_ = 3.58	*0.17*
Years of education *n* (%)	Up to 5 Between 6 and 9 Between 10 and 13 Above 13	2 (16.7%) 3 (25.0%) 1 (8.3%) 6 (50%)	0 (0.0%) 2 (20.0%) 3 (30.0%) 5 (50.0%)	2 (16.7%) 0 (0.0%) 8 (66.7%) 2 (16.7%)	*X*_2_ = 12.64	*0.049*^∗^
Employment situation *n* (%)	Formal job Public job Informal job Unemployed Freelance Temporary job Retired	5 (41.7%) 2 (16.7%) 0 (0.0%) 0 (0.0%) 0 (0.0%) 4 (33.3%) 1 (8.3%)	1 (10.0%) 0 (0.0%) 1 (10.0%) 4 (40.0%) 4 (40.0%) 0 (0.0%) 0 (0.0%)	1 (8.3%) 0 (0.0%) 0 (0.0%) 9 (75.0%) 1 (8.3%) 0 (0.0%) 1 (8.3%)	*X*_2_ = 33.53	*0.001*^∗∗∗^
Marital state *n* (%)	Single Married or Common-law Divorced Widow Not reported	2 (16.7%) 10 (83.3%) 0 (0.0%) 0 (0.0%) 0 (0.0%)	3 (33.3%) 1 (10.0%) 2 (22.2%) 3 (33.3%) 1 (11.1%)	5 (41.7%) 6 (50.0%) 0 (0.0%) 0 (0.0%) 1 (8.3%)	*X*_2_ = 20.3	*0.009*^∗∗^
*Patterns of drug use*						
Amount of daily use Age at onset of drug use (years)		0 (0.0) –	14.8 (6.9) rocks/day 33.4 (9.1)	23.9 (17.0) drinks/day 16.6 (5.0)		
Tobacco use	Yes No	2 (16.7%) 10 (83.3%)	5 (55.6%) 4 (44.4%)	6 (50.0%) 6 (50.5%)	*X*_2_ = 4.15	*0.126*
*Clinical examination*						
FAB^a^ MMSE^a^ HAM-D^b^ HAM-A^b^ Craving scores^b^		13.3 (3.1) 28.0 (2.5)	14.3 (3.1) 28.4 (2.3) 6.5 (2.9) 10.3 (8.4) 5.5 (5.6)	12.1 (2.8) 27.5 (3.6) 4.3 (1.7) 9.3 (8.3) 6.6 (5.8)	*F*(2,31) = 1.3 *F*(2,31) = 0.22	*0.29* *0.80*
**FosB* Expression (Fold change and Ct mean values)*						
Ct mean	Beta-actin	17.03	17.03	16.44		
	*FosB*	25.14	25.34	25.66		
ΔCt		25.14–17.03 = 8.11	25.34–17.03 = 8.31	25.66–16.44 = 9.22		
Fold-change			0.87	0.46		
Expression reduction (−1/fold-change)			−1.15	−2.17		

*Craving scores, 5-items OCDS or OCCS, FAB, frontal assessment battery; MMSE, mini-mental status examination; HAM-D, Hamilton depression; HAM-A, Hamilton anxiety. ^a^, data from two CUD subjects were missing; ^b^, data from four CUD and AUD subjects were missing. ^∗^p < 0.05, ^∗∗^p < 0.01, ^∗∗∗^p = 0.001.*

The inclusion criteria for this study were: (1) male and female patients over the age of 18 years; (2) met criteria for crack-cocaine and alcohol dependence according to the ICD-10 Classification of Mental and Behavioral Disorders and the Diagnostic and Statistical Manual of Mental Disorders, fifth edition, as determined by clinical evaluation; (3) in stable clinical condition with no need for emergency care; (4) able to read, write, and speak Portuguese; and (5) no severe withdrawal signs or symptoms at baseline.

Furthermore, exclusion criteria included: (1) a condition of intoxication or withdrawal due to a substance other than crack-cocaine and alcohol, (2) unstable mental or medical disorder or substance abuse or addiction other than crack-cocaine and alcohol dependence, except nicotine and/or caffeine; (3) diagnosis of epilepsy, convulsions, or delirium tremens during abstinence from crack-cocaine and alcohol.

This study was approved by the Brazilian Institutional Review Board of the Federal University of Espírito Santo (CAAE 19403713.6.0000.5060), Brazil. It was conducted in strict adherence to the Declaration of Helsinki and is in accordance with the ethical standards of the Committee on Human Experimentation of the Federal University of Espírito Santo, Vitória, Brazil.

### Experimental Protocol

Peripheral blood samples (5 ml) were collected from the cubital vein in tubes containing ethylenediaminetetraacetic acid (EDTA). The interval between this blood collection and the isolation of lymphocytes was of the maximum of 3 h. Total RNA was extracted from lymphocytes using the QIAamp Blood Mini Kit^®^ (Qiagen, Germany), and the amount and purity of RNA was determined by spectrophotometry. Aliquots of RNA were subjected to reverse transcribed for complementary DNA (cDNA) using RT^2^ First Strand Kit^®^ (Qiagen, Germany) according to the manufacturer’s protocol in a final volume of 20 μl.

The primers used for amplification of *FosB* and beta-actin genes in real-time PCR reaction were purchased from Qiagen company primer bank.

Real-time quantitative PCR (RT-PCR) was performed using the *ABI PRISM 7500 Sequence Detection Systems^®^* (Applied Biosystems, United States) in combination with SYBR green detection (Qiagen, Germany). The reactions were optimized in a 10 ml reaction volume containing 2 μl cDNA, 5 μl RT^2^ SYBR Green ROX FAST Mastermix^®^ (Qiagen, Germany), 0.4 μl beta-actin (NM_001101.3, Qiagen, Germany) and *FosB* (NM_006732.2, Qiagen, Germany) and 2.6 μl H_2_O. The general PCR condition profile was: Taq polymerase activation at 95°C for 10 min, followed by 40 cycles of denaturing at 95°C for 15 s, annealing at 60°C for 1 min, and extension at 95°C for 15 s. After amplification, a melting curve was acquired to determine the optimal PCR conditions.

### Data Analysis

The Ct mean for *FosB* was subtracted from Ct mean for beta-actin for group control, CUD and AUD, yielding ΔCt. Then the ΔCt from control group was subtracted from ΔCt of CUD and AUD, yielding a ΔΔCt values. Fold-change was found using the formula:

2^−ΔCtAddicted^/2^−ΔCtControl^ and the values expression reduction was found according with Schmittgen and Livak (2008) proceeding the division of −1/calculated fold-change.

### Statistical Analysis

ANCOVA was used to compare quantitative data among groups considering age as covariate and the proportion of Ct values of *FosB* over Ct values of beta-actin (*FosB*/Act) from each subject converted in logarithm scale (Yamada et al., 2013). SPSS Statistics Base 24.0 (SPSS Inc., United States) and GraphPad Prism 7.0 (GraphPad Software Inc., United States) were employed for statistical analysis and graphic presentations, and the *P*-value of less than 0.05 was considered statistically significant.

Effect sizes were calculated using Cohen’s d and corrected by Hedges’s *g*_s_ for between-group comparisons of Log *FosB*/Act values (Lakens, 2013).

## Results

We used RT-PCR to measure mRNA expression levels in human PBLs of CUD and AUD patients in comparison with non-addicted controls.

These groups were well-paired by age and gender, but other socio-demographic characteristics was unequal (Table 1). Schooling was found different among groups (*p* < 0.05) possibly because of higher proportion of middle school degree in AUD patients when compared to higher school degree in control and CUD patients; employment situation was different among groups (*p* = 0.001) as larger proportion of CUD and AUD patients was unemployed and/or working as freelancers and smaller proportion of them was formally employed; and marital state was also different among groups (*p* < 0.01) as higher proportion was married or living in common law in control and AUD groups, whereas CUD patients were mostly single or widow (Table 1). These differences could be expected to be seen in crack-cocaine and alcohol addicted population due to important behavioral and social consequences of these substance use disorders.

Alcohol use disorder and CUD patients used high amount of alcohol drinks and rocks of crack-cocaine per day, respectively (Table 1). More than half of them were tobacco users but not significantly different from control group. No between group differences were found regarding cognitive performance (FAB and MMSE) (Table 1, see Supplementary Material for clinical measurements description). Symptoms of depression in the HAM-D scored into normal range and of anxiety (HAM-A) indicated mild symptoms in both AUD and CUD patients. Craving scores (5-items of OCDS or OCCS) were mild to moderate in both substance use disorder. Mean scores of these clinical measurements are similar to those found in our previous studies in these specific substance use disorders (Klauss et al., 2014; Batista et al., 2015).

**FIGURE 1:**
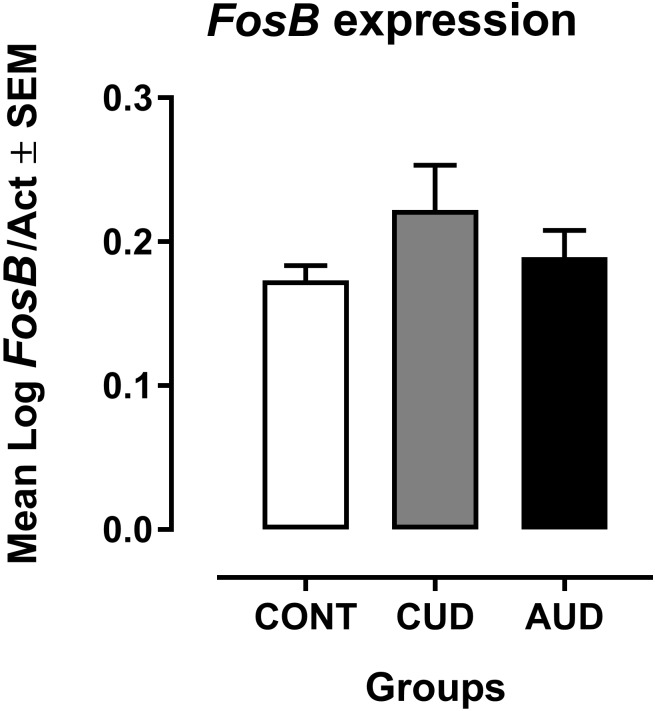
Mean Log *FosB*/Act values from both CUD (*n* = 10) and AUD (*n* = 12) patients compared from the mean of the control group (CONT, *n* = 12). Larger Ct values reflect later threshold cycle, that is, the quantitative cycle in which the target was captured is delayed, meaning that the target is less expressed.

Mean Log *FosB*/Act values from both CUD and AUD patients were slightly larger from the mean found in the control group (Figure 1). However, no statistically significant difference was found in the ANCOVA having age as covariate [*F*(2,30) = 1.002, *p* = 0.379, η^2^ = 0.063]. But, Hedge’s effect size g_s_ was of 0.66 for CUD and of 0.30 for AUD when Log *FosB*/Act was individually compared to controls.

It must be noted that in gene expression, larger Ct values reflect later threshold cycle, that is, the quantitative cycle in which the target was captured is delayed, meaning that the target is less expressed (Schmittgen and Livak, 2008).

In fact, when considering the fold-change analysis, the *FosB* mRNA was less expressed by 1.15-fold in the CUD patient and by 2.17-fold in the AUD patients when normalized to controls (Table 1).

## Discussion

According to “peripheral marker hypothesis,” changes in genetics expression in the brain are reflected in peripheral blood lymphocytes (Roozafzoon et al., 2010). Basing on this theory, our study may constitute the first to demonstre the *FosB* mRNA expression in lymphocytes from peripheral blood, and to show that it is possibly altered in drug addicted patients.

Hedge’s g_s_ of 0.66 for CUD and of 0.30 for AUD indicate, respectively, medium and small effect sizes according to Cohen (1992) in between-groups comparisons, suggesting that although no significant differences were found in the statistical analysis, effects sizes over controls could be meaningful.

Previous studies in rodents indicated that chronic exposure to drugs modulates brain reward regions through the increased of a FosB isoform (delta-FosB) directly measured in reward circuits (Vialou et al., 2012). We found that expression levels of *FosB* mRNA was decreased in peripheral blood of CUD and AUD patients. This reduced *FosB* mRNA expression may reflect the long-term exposition to the drugs as there is experimental evidence that the degree of *FosB* gene induction partially desensitizes with repeated exposure to amphetamine (Alibhai et al., 2007). Besides, Renthal et al. (2008) demonstrated that accumulation of delta-FosB after chronic amphetamine exposure desensitizes c-fos mRNA induction to a subsequent drug dose.

A recent *post-mortem* study showed that the expression of FosB isoforms was downregulated in the hippocampus of addicted patients (Gajewski et al., 2016). Differently, we are here measuring *in vivo* *FosB* gene expression in PBLs.

Levels of *FosB* mRNA expression in PBLs may represent a peripheral marker for molecular changes in substance use disorders and could be useful to follow the after effects of drug dependence treatment, especially those that potentially modify synaptic plasticity such as non-invasive brain stimulation (Cirillo et al., 2017), which has shown to decrease craving and relapses to the drug use (Klauss et al., 2014; Batista et al., 2015). This possibility needs to be carefully investigated.

There are limitations that must be considered. The high complexity and cost of the method with limited budget, and the restricted inclusion and exclusion criteria, have limited the number of subjects included in our samples. We have collected 36 samples from non-addicted controls, 27 from AUD and 17 from CUD patients, but included in the analysis only technically adequate samples. Here we explored the expression of only one gene, but other gene expressions such as of *BDNF* and dopamine receptors are under collection or processing and will be published in the near future.

Patients included in this study were recruited from our major clinical trial registered in ClinicalTrials.gov^1,2^

In summary, *FosB* mRNA expression was detected in lymphocytes from peripheral blood and showed to be mildly reduced in drug addicted patients. Thus, with a great caution because these are preliminary data, it may be suggested that this molecular technique could constitute a potential peripheral marker to measure changes in substance use disorders.

## Author Contributions

All authors have read and approved the manuscript for submission; have made a substantial contribution to the conception, design, gathering, analysis and/or interpretation of data and a contribution to the writing and intellectual content of the article; and acknowledge that they have exercised due care in ensuring the integrity of the work.

## Conflict of Interest Statement

The authors declare that the research was conducted in the absence of any commercial or financial relationships that could be construed as a potential conflict of interest.

## Footnotes

1 https://clinicaltrials.gov/ct2/show/NCT02091284

2 https://clinicaltrials.gov/ct2/show/NCT02091167
