# Supplementary material for: FosB mRNA Expression in Peripheral Blood Lymphocytes in Drug Addicted Patients
Source: Front Pharmacol. 2018 Oct 24;9:1205. doi: 10.3389/fphar.2018.01205 (PMC6207645; doi:10.3389/fphar.2018.01205)
Supplement: Supplementary file 1 [file Data_Sheet_1.PDF]

Supplementary material

Clinical assessments

Craving (five-items from OCDS or OCCS)

Craving was scored with a brief scale composed of 5 items (1, 2, 4, 5, and 13) from the Obsessive-Compulsive Drinking Scale (OCDS) (Anton et al., 1995; Anton et al., 1996; Anton, 2000) for AUD, and from Obsessive Compulsive Cocaine Scale (Vorspan et al., 2012) for CUD, which assesses craving in a narrow sense according to De Wildt et al. (2005). The questions of this brief scale are answered on a scale ranging from 0 to 4, resulting in a total score between 0 and 20.

Frontal Assessment Battery (FAB)

The executive function domains were explored using the FAB (Dubois et al., 2000). Six different domains were analyzed: 1) conceptualization; 2) mental flexibility; 3) motor programming; 4) sensitivity to interference; 5) inhibitory control; and 6) autonomy. Each of these items is scored from 0 (zero) to a maximum of 3. Thus, the maximum score of FAB is 18. A single well-trained examiner administered this assessment. The instrument was carefully explained and pre-trained shortly before its full application.

Mini Mental Status Examination (MMSE)

An adapted version of the MMSE in Portuguese was used, which includes an 11-item examination of five areas of cognitive function: orientation, registration, attention and calculation, recall, and language. The maximum score that could be achieved was 30, while a mean score between 23 and 26 or between 26 and 29 would be expected according to age and educational level (Crum et al., 1993) of the patients. The instrument was also explained and pre-trained shortly before its full application.

Hamilton Depression Rating Scale (HAM-D)

The severity of depression symptoms was analyzed by a multiple-choice questionnaire. This instrument assesses the severity of depression symptoms such as low mood, insomnia, agitation, anxiety and weight loss (Hamilton, 1960). The examiner must choose between the possible answers to each question by interviewing the patient and by observing the patient's symptoms. Each question has between 3 and 5 possible answers that increase in severity. In the original scale, the first 17 questions contribute to the total score, while questions 18 to 21 provide additional information about depression (e.g., diurnal variation, paranoid symptoms), but are not included in the total score of the scale.

Hamilton Anxiety Rating Scale (HAM-A)

The severity of anxiety symptoms was analyzed via a structured multiple-choice questionnaire (Hamilton, 1959). The scale consists of 14 items, each defined by a series of symptoms, and measures both psychic anxiety (e.g., mental agitation and psychological distress) and somatic anxiety (e.g., physical complaints related to anxiety).

References

Anton RF (2000) Obsessive-compulsive aspects of craving: development of the Obsessive Compulsive Drinking Scale. Addiction 95 Suppl 2:S211-217.
Anton RF, Moak DH, Latham P (1995) The Obsessive Compulsive Drinking Scale: a self-rated instrument for the quantification of thoughts about alcohol and drinking behavior. Alcohol Clin Exp Res 19:92-99.
Anton RF, Moak DH, Latham PK (1996) The obsessive compulsive drinking scale: A new method of assessing outcome in alcoholism treatment studies. Arch Gen Psychiatry 53:225-231.
Crum RM, Anthony JC, Bassett SS, Folstein MF (1993) Population-based norms for the Mini-Mental State Examination by age and educational level. JAMA 269:2386-2391.
de Wildt WA, Lehert P, Schippers GM, Nakovics H, Mann K, van den Brink W (2005) Investigating the structure of craving using structural equation modeling in analysis of the obsessive-compulsive drinking scale: a multinational study. Alcohol Clin Exp Res 29:509-516.
Dubois B, Slachevsky A, Litvan I, Pillon B (2000) The FAB: a Frontal Assessment Battery at bedside. Neurology 55:1621-1626.
Hamilton M (1959) The assessment of anxiety states by rating. Br J Med Psychol 32:50-55.
Hamilton M (1960) A rating scale for depression. J Neurol Neurosurg Psychiatry 23:56-62.
Vorspan F, Bellais L, Romo L, Bloch V, Neira R, Lepine JP (2012) The Obsessive-Compulsive Cocaine Scale (OCCS): a pilot study of a new questionnaire for assessing cocaine craving. Am J Addict 21:313-319.
